# The Recombinant Luciferase of the Fungus *Neonothopanus nambi*: Obtaining and Properties

**DOI:** 10.1134/S1607672921010051

**Published:** 2021-03-10

**Authors:** A. Yu. Gorokhovatsky, T. V. Chepurnykh, A. S. Shcheglov, Yu. A. Mokrushina, M. N. Baranova, S. A. Goncharuk, K. V. Purtov, V. N. Petushkov, N. S. Rodionova, I. V. Yampolsky

**Affiliations:** 1 Shemyakin–Ovchinnikov Institute of Bioorganic Chemistry, Russian Academy of Sciences, Moscow, Russia; 2 Institute of Biophysics, Federal Research Center “Krasnoyarsk Scientific Center of the Siberian Branch of the Russian Academy of Sciences”, Krasnoyarsk, Russia

**Keywords:** bioluminescence, luciferase, nnLuz, *Neonothopanus nambi*, heterologous expression, *Pichia pastoris*

## Abstract

A key component of the recently described bioluminescent system of higher fungi is luciferase, a new class of proteins. The properties of fungal luciferase and their relationship with its structure are interesting both for improving autoluminescent systems already created on its basis and for creating new ones. Therefore, it is extremely important to understand the spatial structure of this protein. We have performed heterologous expression and purification of *Neonothopanus nambi* luciferase, obtained a protein suitable for subsequent crystallization, and also determined some biochemical properties of the recombinant luciferase.

Bioluminescent systems are widely used both for research purposes and for drug development and diagnostics [1–6]. The recently clarified bioluminescent system of fungi is a promising tool for biomedical research [7]. On its basis, autonomously luminescing yeast [8] and plants [9] have already been created. However, the key component of this system, luciferase (nnLuz), remains poorly understood. The lack of homology with other enzymes does not allow modeling the spatial structure of this protein, and the difficulty of its isolation from a natural source prevents obtaining a sufficient amount of this protein for crystallization and characterization of its properties. Thus, obtaining recombinant *N. nambi* luciferase is currently a relevant task.

At the first stage of the study, we compared the luciferase activity of *N. nambi* expressed in three standard systems: *E. coli*, yeast *Pichia pastoris*, and HEK293T human cell line. The yeast *P. pastoris* demonstrated the maximum luminescence in response to the addition of fungal luciferin. Therefore, for further experiments, we decided to use the yeast strain, producing luciferase with a sequence of six histidine residues at the C-terminus of the protein. Yeast was cultured according to the standard procedure using the GS115 strain [10].

Yeast biomass was lysed in 100 mM Na-phosphate buffer (pH 7.0) containing 100 mM NaCl, 5 mM EDTA, 10 mM 2-mercaptoethanol, and 1 mM PMSF in a high-pressure homogenizer (600 bar, IKA HPH). The lysate was centrifuged (8000 *g*, 30 min) at 4°C and the membrane fraction containing luciferase was pelleted by ultracentrifugation (150 000 *g*, 90 min) at 4°C. To select the conditions for luciferase solubilization, the membranes were suspended in 50 mM HEPES-Na buffer (pH 8.0) containing 500 mM NaCl, 20% glycerol (buffer A), and various detergents at 4°C overnight (Fig. 1). Almost all of the detergents showed approximately the same ability to extract luciferase, except for the detergent OG (octyl glycoside). For preparative extraction, we used DDM at a concentration of 10 mM, due to its availability and good applicability for chromatography with UV detection. All chromatographic experiments were performed in the cold. The detergent-solubilized membrane fraction was loaded onto a column packed with the TALON sorbent for metal affinity chromatography equilibrated with buffer A with 0.02% DDM, and the column was washed with the same buffer. Then, the column was washed with 20 mM MES-Na, 0.5 M NaCl, and 10% glycerol buffer (pH 6.2, buffer B) containing 0.02% DDM. Adsorbed luciferase was eluted with buffer B containing 0.1% DDM and 200 mM imidazole. The fractions with the highest luminescence activity were loaded onto a column with Sephacryl S-300 in 50 mM Na-phosphate buffer (pH 7.0) containing 150 mM NaCl and 0.04% DDM for gel filtration. As a result, a recombinant *N. nambi* luciferase with a purity of more than 95% (Fig. 2) and a yield of 10 mg per liter of *P. pastoris* culture was obtained.

**Fig. 1.**
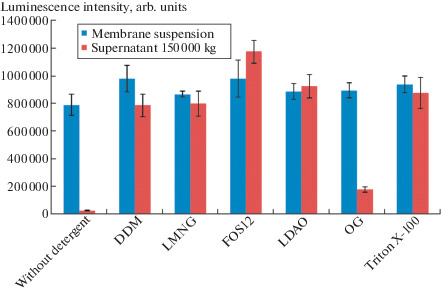
Solubilization of nnLuz luciferase from *P.* *pastoris* membranes suspended in buffer (see text) with addition of various detergents at a concentration of 10 mM (100 mM for OG) for 16 h at 4°C. After centrifugation (150 000 *g*, 90 min) at 4°C, the bioluminescence activity of luciferase in the supernatant was measured in 200 mM Na-phosphate buffer (pH 8.0) containing 500 mM Na_2_SO_4_, 0.1% DDM, and 50 μM fungal luciferin. Data are represented as the mean value ​​± standard deviation.

**Fig. 2.**
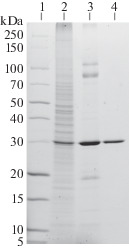
Electrophoresis under denaturing conditions of fractions during isolation of the recombinant *N. nambi* luciferase. Designations: 1—molecular weight markers; 2—membrane fraction of *P. pastoris* cells; 3—fraction containing the recombinant *N. nambi* luciferase after metal chelate chromatography; 4—fraction containing the recombinant *N. nambi* luciferase after gel filtration chromatography. The gel was stained with Coomassie Brilliant Blue G250.

During gel filtration on a Superdex 200 column in the presence of DDM detergent micelles, luciferase is eluted as a single peak corresponding to a molecular weight of about 60 kDa. On the one hand, luciferase can be a dimer, because the calculated monomer mass determined from the amino acid sequence is 31.4 kDa. However, given the size of the DDM micelle (40–70 kDa) and gel filtration conditions, luciferase is more likely to be a detergent-bound monomer, although the exact stoichiometry of this complex remains unknown.

The luciferase preparation obtained by gel filtration was studied by circular dichroism spectroscopy. The spectrum was recorded in 20 mM Na-phosphate buffer containing 100 mM NaCl and 0.04% DDM (pH 7.4) at 5°C on a J-810 spectropolarimeter (JASCO, Japan) at a protein concentration of 11 μM in a cell 0.01 cm thick. Data were processed using the CONTINLL software (CDPro package) and a set of reference spectra (SMP56). The results of the analysis of the obtained spectrum are presented in Table 1. The presence of beta-structures and alpha-helical regions presented in Table 1 indicates that the purified luciferase preparation with a high probability contains the protein with the native structure.

**Table 1.** Analysis of the obtained circular dichroism spectrum

Number of aa	Alpha-helix, %	Beta structure, %	Turn, %	Unordered, %	NRMSD
275	21.5	26.8	22.5	29.1	0.05

Luciferase activity in vitro strongly depends on the presence of detergents in the medium (Fig. 3). We compared the bioluminescence activity of the recombinant nnLuz in the presence of Tween-20, Triton X-100, NP-40, digitonin, DDM, FOS-12, and CHAPS. Different detergents contribute to the luminescence reaction to varying degrees. The highest luciferase activity was observed in the presence of FOS-12 (*n*-dodecylphosphocholine). However, we showed that, during storage in the presence of the detergent FOS-12, the purified nnLuz lost its activity, whereas during storage in the presence of DDM, no loss of luciferase activity was observed. Therefore, the purification and measurement of luciferase activity was also performed in the presence of DDM in order to prevent the formation of mixed micelles during measurements.

**Fig. 3.**
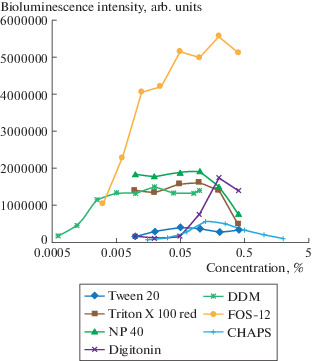
Effect of detergents on luciferase bioluminescence. The reaction was performed in 200 mM Na-phosphate buffer (pH 8.0) containing the indicated concentration of the detergent and 50 μM of fungal luciferin. The measurements were performed for 30 s. The integrated light intensity values ​​are shown.

The optimum pH for the bioluminescence reaction is 8.0 (Fig. 4a). The recombinant luciferase is a temperature-sensitive enzyme: incubation at 30°C for 10 min almost completely inactivated the enzyme (Fig. 4b).

**Fig. 4.**
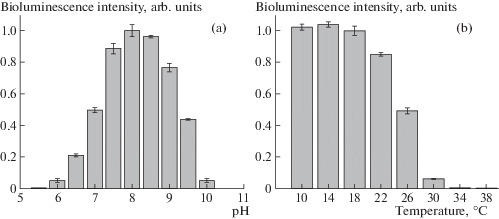
pH-dependence (a) and thermal stability (b) of *N. nambi* luciferase bioluminescence. The luminescence measurement was carried out after incubation for 10 min at the indicated temperature in 100 mM Na-phosphate buffer (pH 7.0). The reaction was performed in 100 mM buffer at the indicated pH containing 0.1% DDM and 50 μM fungal luciferin at room temperature. The light was integrated for 60 s. Data are represented as the mean value ​​± standard deviation.

The Michaelis–Menten constant was determined for the luciferin oxidation reaction catalyzed by the recombinant *N. nambi* luciferase. The reaction was performed at a luciferase concentration of 50 nM in 200 mM Na-phosphate buffer (pH 8.0) containing 500 mM Na_2_SO_4_ and 0.1% DDM at room temperature; the concentration of fungal luciferin was varied from 2 nM to 50 μM. The initial bioluminescence intensity values were approximated by the Michaelis–Menten function using the Origin software package. *K*_M_ was 1.09 ± 0.06 μM.

Thus, in this study, we obtained and characterized the recombinant *N. nambi* luciferase. The developed technique for isolating this enzyme will be further used to accumulate sufficient amounts of the protein for crystallization and study of the spatial structure of luciferase by NMR.
